# Comprehensive mutational analysis of the sequence–function relationship within a viral internal ribosome entry site

**DOI:** 10.1093/nar/gkaf445

**Published:** 2025-05-27

**Authors:** Sabrina G Grunseich, Scott A Strobel

**Affiliations:** Department of Chemistry, Yale University, New Haven, CT 06511, United States; Institute of Biomolecular Design and Discovery, West Haven, CT 06516, United States; Department of Chemistry, Yale University, New Haven, CT 06511, United States; Institute of Biomolecular Design and Discovery, West Haven, CT 06516, United States; Department of Molecular Biophysics and Biochemistry, Yale University, New Haven, CT 06510, United States

## Abstract

The cricket paralysis virus (CrPV) intergenic region internal ribosome entry site (IRES) binds to the ribosome without the need for any initiation factors. Their length, simple mechanism, and ability to function in diverse cell-free systems make CrPV-like IRESs useful tools to study the mechanism of translation and to express proteins. We report the use of a RelE-based next-generation sequencing method, termed SMARTI (sequencing-based mutational analysis of RNA translation initiation), to quantitatively determine the function of over 81 000 single and double mutants of CrPV IRES. The result is a comprehensive mutational database that serves as a consensus sequence-like analysis of IRES function. We have given particular attention to the sequence requirements within the three pseudoknots of the IRES element. The data indicate that each pseudoknot contains positions that are modifiable and mutation may even enhance IRES function through pseudotranslocation. CrPV IRES must balance being stable and dynamic as it forms the structure and ribosomal contacts required for translation initiation. Helical regions, especially in the transfer RNA-mimicking domain, are areas where flexibility may be especially beneficial. Moreover, we demonstrated that this high-throughput method is compatible with eukaryotic extract, providing an avenue for studying diverse eukaryotic RNA elements and for engineering sequences for protein expression.

## Graphical Abstract

**Graphical Abstract:**
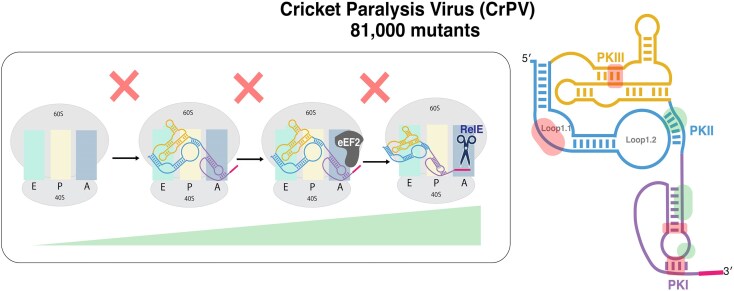

## Introduction

Viruses have evolved mechanisms to manipulate translation at many stages of the process, including initiation, elongation, termination, and recycling [1]. Internal ribosome entry sites (IRESs) are *cis*-acting viral RNA elements used to manipulate initiation. They allow some viruses to recruit host translation machinery without the use of all canonical factors [2]. Using these structured RNAs, the virus can continue translating its own protein products even while cap-dependent translation is repressed by the host fighting the infection [3].

Viral IRESs are divided into distinct types based on their secondary structure, length, protein factors required, and mechanism of action [3, 4]. IRESs identified in the intergenic regions (IGRs) of the *Dicistroviridae* family have a specific secondary structure, are usually <200 nucleotides in length, and use the simplest mechanisms compared to other viral IRESs [5]. These IRESs, recently termed Type 6 IRESs, initiate translation from a non-AUG start codon, do not use initiator Met-tRNA, and do not require any initiation factors [6, 7].

**Figure 1.**
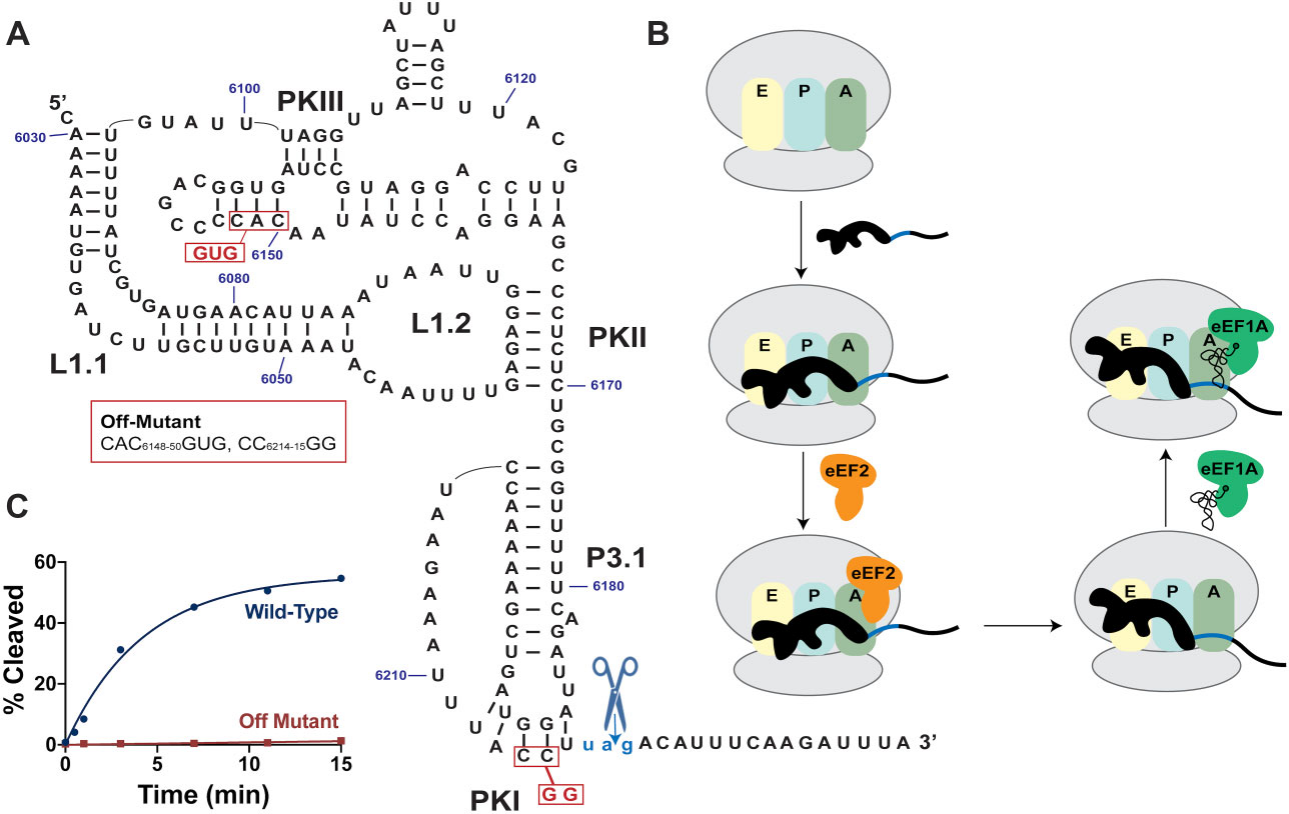
(**A**) Secondary structure diagram of CrPV IGR IRES sequence. The first translatable codon has been mutated, GCU_6217–6219_UAG, for efficient RelE cleavage. (**B**) Model of translation initiation mechanism used by CrPV IGR IRES. The black shape is CrPV IGR IRES and the yellow, teal, and green ovals are the respective E, P, and A sites of an 80S ribosome, gray ovals. After binding across all decoding sites, CrPV IGR IRES is pseudotranslocated by eEF2, which places its first translatable codon, blue, into the A-site. tRNA delivery by eEF1A can then occur. (**C**) Gel-based RelE cleavage experiments using the wild-type sequence and an off-mutant described in panel (A).

One of the best-studied Type 6 IRESs is from the cricket paralysis virus (CrPV). The CrPV IRES is 190 nucleotides in length and is comprised of three domains (Fig. 1A). Domains 1 and 2 make up the ribosome-binding domain and contain pseudoknot II and pseudoknot III (PKII and PKIII). These pseudoknots play essential roles in orienting the IRES for its ribosomal binding contacts [8]. Domain 3 folds independently from its counterparts and serves as a transfer RNA (tRNA) mimic with pseudoknot I (PKI) imitating the codon/anticodon interaction [9]. CrPV IRES can bind to the 40S subunit followed by a stabilizing conformational change and subsequent recruitment of the 60S subunit [10, 11]. It does this without the need for initiation factors. The RNA structure is bound across all three decoding sites, with PKI occupying the A-site [12, 13]. CrPV undergoes an eEF2-mediated pseudotranslocation step to place the first translatable codon in the A-site and generate an open A-site for tRNA delivery (Fig. 1B) [14, 15]. Following pseudotranslocation, tRNA delivery by eEF1A and another round of translocation occur before typical elongation cycles commence [10].

Roles for each domain of the CrPV IRES were established by mutating several bases and whole helices. That work enabled directed mutational screens and identified key nucleotides [8, 9, 16–22]. Single nucleotides within loop 1.1 have been identified as key positions for 60S binding [18]. Single mutations to the tRNA-mimicking domain, specifically in the PKI containing loop, demonstrated that interactions between those positions were important for IRES function [17].

There are limited published examples of CrPV-like IRESs [5, 20, 23–26]. For this reason, a consensus sequence generated by bioinformatics may not fully reveal the relationship between IRES sequence and function. However, an understanding of the sequence requirements at the individual residue level would be useful for synthetic biology applications. Its length, simple mechanism, lack of a need for initiation factors, and ability to function in diverse cell-free systems make CrPV-like IRESs useful tools for protein production applications [27–29]. Therefore, an analysis of this system is warranted to understand the CrPV IRES sequence requirements and to improve the utility of this IRES as a biological tool.

Recent studies have investigated regulatory RNA function using high-throughput methods [30–35]. To perform a comprehensive mutational analysis of the CrPV IRES, we implemented the high-throughput assay, SMARTI (sequencing-based mutational analysis of RNA translation initiation) [34]. SMARTI utilizes the endonucleolytic activity of RelE, a bacterial endonuclease that binds and cleaves ribosome-bound messenger RNA (mRNA) in the A-site, to detect translation initiation [36, 37]. Specific cleavage between the second and third nucleotides in the A-site codon allows for a sensitive, sequence-specific measurement of mRNA–ribosome loading.

Here, we show that this assay is versatile and can be used to study translationally active eukaryotic RNAs in unpurified cell extract. This high-throughput method provides a comprehensive mutational analysis at single-nucleotide resolution. We have identified positions in the CrPV IGR IRES sequence that are essential for specific steps in its mechanism of translation. These data provide clarity about the dynamics of the CrPV IGR IRES structure that can be leveraged to better understand the function of other Type 6 IRESs. Moreover, data made available from this method create an opportunity for optimizing RNA sequences for their use as engineered biological tools.

## Materials and methods

### Design of IRES constructs

Mutant libraries of the CrPV IGR IRES were created from the wild-type sequence (NC_003924.1). The wild-type CrPV IGR IRES includes nucleotides 6007–6234 with the following changes: a G was added on the 5′ end to improve transcription yield; the mutation GCU_6217–6219_UAG was made to accommodate RelE’s *in vitro* sequence sensitivity and enable efficient and reliable RelE cleavage; and the mutation AA_6232–6233_UU was made so that full-length and cleaved products had the same terminal triplet that would remove possible ligation bias. Three mutant libraries were generated including a single mutant library that encompassed the entirety of the IRES (6029–6216), a double mutant library focused on the ribosome binding domains (6054–6073, 6096–6170), and an additional double mutant library encompassing multiple regions including pseudoknot I (6029–6036, 6043–6053, 6062–6067, 6074–6084, 6089–6096, 6165–6216). Libraries were made using doped oligonucleotides with mutation frequencies of 1.8% (0.6% for each nucleotide alternative) and 2.1% (0.7% for each nucleotide alternative) for single and double mutant libraries, respectively.

### Transcription template preparation

DNA oligonucleotides were ordered from Keck Oligo Synthesis Resources at Yale University and used to generate transcription templates via overlap extension using SuperScript II RT (Invitrogen) and nucleotides 6116–6135 as the overlapped region. The extended product was separated on a 2% Agarose E-Gel (Invitrogen) and purified using GeneJET Gel Extraction Kit (Thermo Fisher Scientific). T7 promoter sequence was added by polymerase chain reaction (PCR) followed by template amplification by PCR. PCR was performed using Phusion High-Fidelity DNA Polymerase (New England Biolabs) and purified using QIAquick PCR Purification Kit (Qiagen).

### RNA preparation and labeling

*In vitro* transcription reactions contained 80 mM HEPES–NaOH (pH 7.5), 1 mM spermidine, 120 μg/ml bovine serum albumin, 10 mM dithiothreitol (DTT), 40 mM MgCl_2_, 5 mM of each NTP, 16 ng/μl DNA template, 1 U/ml inorganic pyrophosphatase (Sigma–Aldrich), and in-house purified T7 RNA polymerase. After a 4-h incubation at 37°C, RNA was purified by denaturing polyacrylamide gel electrophoresis (PAGE) and the RNA band was excised and crushed before soaking in 300 mM NaOAc at 4°C overnight. Gel pieces were subsequently filtered and the RNA was ethanol precipitated at −20°C and pelleted by centrifugation at 9250 rpm at 4°C followed by resuspension. For 5′ radiolabeling, RNA was dephosphorylated with Antarctic Phosphatase (New England Biolabs) and labeled with ^32^P-γ-ATP (Perkin Elmer) using T4 PNK (New England Biolabs). Radiolabeled RNA was purified using Microspin G-50 columns (Cytiva).

### RelE preparation

Wild-type RelE was purified as previously described [38].

### Translation initiation and RelE cleavage

RNA was refolded in 1× Buffer A (20 mM Tris, pH 7.5, 100 mM KOAc, 2.5 mM MgCl_2_, 2 mM DTT, 250 μM spermidine) by incubation at 65°C for 3 min in a heat block followed by slow cooling the block and RNA on the benchtop until reaching room temperature. Translation initiation solutions were assembled as 50% (v/v) wheat germ extract (Promega), 120 mM KOAc, 0.8 U/μl RNaseOUT (Invitrogen), 1.4 mM MgCl_2_, 4 mM spermidine, and 5 μM RelE. The solution was incubated in a thermocycler at 37°C for 2 min at which point 0.25 pmol refolded RNA was added and the reaction was incubated at 37°C for various times from 2 min to 1 h. Reaction components were separated with addition of 40× volumes of water and immediate centrifugation through a 0.5 ml 50K Amicon (Millipore) for 2.5 min. Four hundred ten microliters of water was then added to the Amicon and centrifuged again. This was repeated an additional time. The 0 min time point was performed by incubating the translation initiation reactions with RNA in the absence of RelE at 37°C for 2 min. Then, reaction components were separated with water, RelE was added, and the Amicon centrifugations were performed. Following the centrifugations, all samples were then purified using the Oligo Clean and Concentrator Kit (Zymo). Reactions in rabbit reticulocyte lysate were performed on a longer timescale, 5 h, but otherwise performed and purified identically using the following reaction composition: 30% flexi rabbit reticulocyte lysate (Promega), 120 mM KCl, 0.8 U/μl RNaseOUT (Invitrogen), 2 mM DTT, 1.4 mM MgCl_2_, 4 mM spermidine, and 0.25 pmol refolded RNA. Gel-based assays differed in the following ways: the refolding solution additionally contained a trace amount of radiolabeled RNA; reactions were quenched with 3× volumes of FLB (25 mM ethylenediaminetetraacetic acid and <0.1% xylene cyanol); and reaction products were separated by denaturing 10% PAGE, and visualized and analyzed with a Typhoon Imager (Cytiva) and ImageQuantTL software (Cytiva).

### Preparation of RNA for high-throughput sequencing

T4 PNK (New England Biolabs) was used to heal the 2′,3′-cyclic phosphate left by RelE cleavage and reestablish the 3′OH. The T4 PNK mastermix was added directly to each RNA sample and incubated at 37°C for 1-h at which point reactions were purified using the Oligo Clean and Concentrator Kit (Zymo). Ligation of a pre-adenylated DNA adaptor (/5rApp/NNNNNCTGTAG GCACCATCAAT/3ddC/; ordered from IDT), reverse transcription off of the ligated adapter to generate complementary DNA, degradation of RNA, and PCR were performed as previously described in [34] with the following changes: ligation was performed without beads; purification of ligation and PCR reactions were performed using CleanNGS SPRI beads (Bulldog Bio) with a 1.5× ratio and 1× ratio, respectively. DNA concentration of each time point sample was determined via Qubit and mixed together at equal ratios before submitting to the Yale Center of Genome Analysis (YCGA) for sequencing.

### High-throughput sequencing

Samples from independent replicates using each of the libraries in wheat germ (the single mutant library, ribosome-binding double mutant library, and pseudoknot I double mutant library) were sequenced by the YCGA on a NovaSeq X Plus sequencing system (2 × 150 bp). Demultiplexing was performed by the YCGA.

### Analysis of sequencing results

Analysis proceeded similarly to [34]. The constant regions on the 5′ and 3′ of ends of the IRES were removed using CutAdapt. The remaining sequences were aligned to the wild-type CrPV IRES sequence using Bowtie 2. Custom Python scripts previously reported were used to determine the fraction of RNA cleaved for all variants with zero to two mutations at each time point [31]. Sequences were considered cleaved if they ended at precisely nucleotide position 6218 consistent with RelE cleavage between the second and third nucleotides in the A-site. Full-length sequences were any sequences terminating at position 6219 or later where RelE did not cleave the RNA. The numbers of full-length and cleaved reads were then counted for each variant to calculate the percentage of cleaved RNA. The data were fit with the one phase association model to assess pseudo-first-order association in R:


\begin{eqnarray*}
Y = {{Y}_0} + {\rm amplitude}*\left( {1 - {{{\rm e}}^{ - kx}}} \right),
\end{eqnarray*}

where *Y* is the percent cleaved, *Y*_0_ is the percent cleaved at time 0, amplitude is the difference between *Y*_max_ and *Y*_0_, *k* is the combined rate constant, and *x* is the time. Standard deviations of all fit values were calculated as previously described [31]. Curves were plotted and visualized in Prism 9. Amplitude data used to generate figures and tables were restricted to those with a standard deviation of <10 percentage points. For the single mutant amplitude figure, amplitudes that could not be fit were calculated by the difference between the first and last time points. Rate constant fit data used to make figures and tables are for curves with an amplitude >5% and with *k* values <0.7 min^−1^ with a standard deviation of less than the value. Average fold changes were calculated by dividing the mutant value by the wild-type value followed by averaging each fold change.

### Dual-luciferase translation assays

A plasmid containing the coding sequence for renilla luciferase (Rluc) as well as the sequence for firefly luciferase (Fluc) under CrPV IGR IRES control was used for the dual-luciferase assays. Mutations were made to this plasmid, pCrPV1-1 [22], using QuikChange II (Agilent). Mutations were verified by Sanger sequencing through the Keck DNA Sequencing Core at Yale University and by whole-plasmid sequencing through Plasmidsaurus. The region containing the T7 promoter sequence through the Fluc coding region was PCR amplified. PCR product was verified by 2% Agarose E-gel (Invitrogen) and 20 μl of this reaction was used directly in 50 μl transcription reactions using the MEGAscript T7 Transcription Kit (Invitrogen). Transcription reactions were purified using the Monarch RNA Cleanup Kit (New England Biolabs). RNA concentration was determined by NanoDrop and RNA integrity was determined by 1% Agarose E-gel (Invitrogen). Translation assays were performed using wheat germ extract (Promega) or Flexi Rabbit Reticulocyte Lysate System (Promega) in the presence of 150 mM KOAc and 1 μg of purified RNA with a final reaction volume of 25 μl. Reactions were incubated in a thermocycler at 30°C for 90 min. Then, 20 μl of each reaction were used with the Dual-Luciferase Reporter Assay System (Promega) and BioTek Synergy4 plate reader. For each mutant, three independent replicates were performed and the average ratio of Fluc/Rluc was calculated. Average ratios were normalized to wild type and error was propagated.

### DNA oligonucleotides and chemicals

All DNA oligos were synthesized by the W.M. Keck Oligonucleotide Synthesis Facility at Yale University unless noted otherwise.

## Results and discussion

### Monitoring translation initiation by CrPV IRES using RelE cleavage

CrPV IRES binds to the ribosome initially with its PKI in the A-site. It requires an eEF2-mediated pseudotranslocation event in order to move PKI and place the first codon in the A-site (Fig. 1B).

RelE is a bacterial endonuclease that binds and cleaves ribosome-bound mRNA in the A-site and has no endonucleolytic activity in the absence of ribosomes [39]. Specific cleavage between the second and third nucleotides in the A-site codon allows for a sensitive, sequence-specific measurement of mRNA–ribosome binding [37]. We have previously shown that RelE cleavage readout can be adapted to a high-throughput approach [34, 36]. Given that the pseudotranslocation event places the first codon of CrPV in the A-site, RelE cleavage can and has been used to locate the IRES in the A-site [7, 22]. Further, this useful readout of IRES initiation can allow simultaneous quantitative analysis of thousands of individual IRES mutants.

To ensure that CrPV IRES would be amenable to a RelE-based method, we first performed a gel-based analysis of CrPV IRES in wheat germ extract [40]. CrPV IGR IRES was incubated in extract for various amounts of time during which RNA that bound to the ribosome was cleaved by RelE in the A-site. Importantly, based on the CrPV mechanism, cleavage would only occur after a successful binding event and pseudotranslocation when the first codon is in the A-site (Fig. 1A and B) [10]. Eukaryotic translation termination factor 1 (eRF1) is present in the extract and its binding to the ribosome would similarly require the open A-site after eEF2 pseudotranslocation [12, 41]. However, RelE cleavage is fast (>100 s^−1^) and likely outcompetes eRF1 for A-site binding, allowing us to view IRES loading onto the ribosome by RelE cleavage [38]. The wild-type CrPV IRES was cleaved by RelE in wheat germ extract with an amplitude of 55% (Fig. 1C). A mutant known to be inactive (CAC_6148–6150_GUG and CC_6214–6215_GG) remained uncleaved when incubated with RelE in wheat germ for the same duration (Fig. 1A and C) [8]. This is consistent with the expectation that RelE only cleaves RNAs bound to the A-site. Most importantly for the current study, it establishes that RelE can be used as an indication of ribosome loading by CrPV IRES in an unpurified translation system.

### High-throughput mutational analysis of CrPV IGR IRES

**Figure 2.**
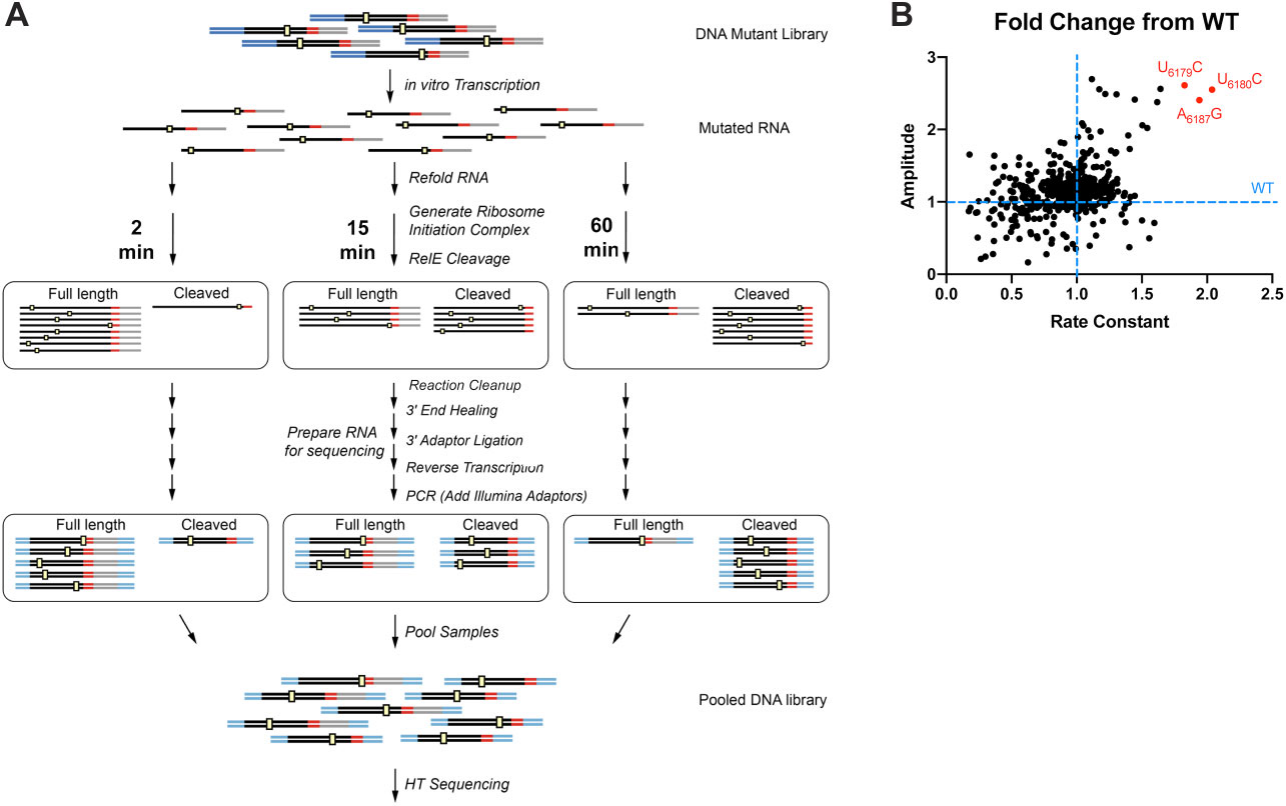
(**A**) Schematic of high-throughput sequencing-based mutational analysis of RNA translation initiation, adapted from [34]. (**B**) Plot of average fold change in amplitude versus average fold change in rate constant of single mutants compared to wild type from all replicate experiments.

We used SMARTI to simultaneously and quantitatively study thousands of CrPV IGR IRES mutants (Fig. 2A) [34]. This high-throughput mutational assay generates ribosome-loading data for thousands of single and double mutants in a single experiment, with binding time courses generated for each mutant.

SMARTI leverages RelE cleavage as a readout of ribosome loading. Similar to the gel-based approach, the IRES RNA is incubated in eukaryotic wheat germ extract with RelE. RelE only cleaves those RNAs that are bound to the ribosome within the A-site. Following the cleavage reactions, the RNA is reverse transcribed and prepared for next-generation sequencing with the addition of time point-specific barcodes (Fig. 2A). The RelE-induced length difference between full-length (unbound) and cleaved (ribosome-bound, pseudotranslocated, and RelE cut) reads is used to determine the percent cleaved at each time point for all 567 single mutants and 80 631 double mutants in the libraries. This length difference is extracted from the sequencing results, allowing for quantitative ribosome-loading data for each mutant in wheat germ. As we do not expect that the rate of RelE cleavage is dependent on the IRES sequence, comparisons between mutants allow us to assess their relative activities. We replicated a portion of the wheat germ SMARTI results in rabbit reticulocyte lysate using single mutants in the PKI library. In this single experiment, we observed similar trends and broad agreement across mutants that were cleaved more efficiently (Supplementary Fig. S1A and B).

We created an RNA mutant library for 188 of the 190 residues in the CrPV IRES encompassing nucleotides 6029–6216 that includes comprehensive point mutations but does not include insertions or deletions. We performed *in vitro* transcription from doped template DNA to maximize for single mutants. To reduce the sequencing reads required to view double mutants, we generated two additional libraries encompassing either the ribosome-binding domains or the tRNA-mimicking domain.

Percent cleaved data were fit to a one-phase association model from which the amplitude and a combined rate constant were extracted (Supplementary Fig. S2). These values were determined for each mutant and compared with wild type (Fig. 2B). The amplitude in our experiment represents the reaction end point after 1-h. This reflects the ability of certain IRES sequences to remain properly folded and outcompete other mutants, and extract factors for ribosomal binding.

Using SMARTI, the wild-type IRES produced an amplitude of 18% ± 4% after a 1-h incubation in wheat germ with RelE. The lower amplitude as compared with the gel-based experiment is similar to what was observed for other systems when transitioning from gel-based to sequencing-based methods [34, 35]. Furthermore, this depression in amplitude is expected to impact all sequences unbiasedly as they are treated together in the same tube and contain the same 5′ and 3′ ends.

We observed a large range in amplitude values across single mutants, 0%–50%, with the most improved mutants achieving an amplitude almost three-fold that of wild type. These mutant changes in amplitude were reproducible (Supplementary Fig. S3A–E). Among the 80 631 double mutants, 8% of mutants resulted in amplitudes of <5% cleaved in both replicate experiments. This low amplitude was interpreted as being nonfunctional. Forty-five percent of double mutants had amplitudes >5% cleaved and could be fit with low standard deviation (10 percentage points). Within this subset of double mutants, 66% showed low standard deviation (5 percentage points) between replicates. Double mutant amplitudes ranged from 0% to 74% cleaved.

The observed rate constant represents the rate at which each sequence can achieve ribosome binding and pseudotranslocation (Supplementary Fig. S3F–J). Across single mutants, the fold change in rate constant ranged from 0.2- to 2.0-fold that of wild type. Among the 23 779 double mutants that showed reliable amplitude between replicates, 62% produced changes in rate constant that met our screening criteria and showed low standard deviation between replicates. Among these double mutants, the fold change in rate constant from wild type spanned from 0.1 to 3.7. Data from two previous studies, which used purified translation machinery to study the kinetics of CrPV IGR IRES translation, showed that pseudotranslocation occurs at a rate that is one and two orders of magnitude slower than 40S and 60S binding, respectively, at the ribosome concentration expected in our wheat germ reactions [10, 12, 42–44]. This suggests that our observed rate constant describes the pseudotranslocation event unless a mutation causes ribosome binding to become rate limiting. However, this is additionally complicated by the instability of the pseudotranslocated IRES and the tendency of the IRES to back-translocate in the absence of tRNA [12, 44]. Additionally, other IGR IRES classes have been shown capable of binding directly to the P-site. It is possible, but unlikely, that point mutations in our library could confer this ability to CrPV. In that case, our data would be unable to differentiate between direct P-site binding and P-site occupation after pseudotranslocation from the A-site. Given the complexity of this system, we are not able to assign the source of rate or amplitude effects for any particular mutant.

### Comprehensive analysis recapitulates functional effects of known mutations

**Figure 3.**
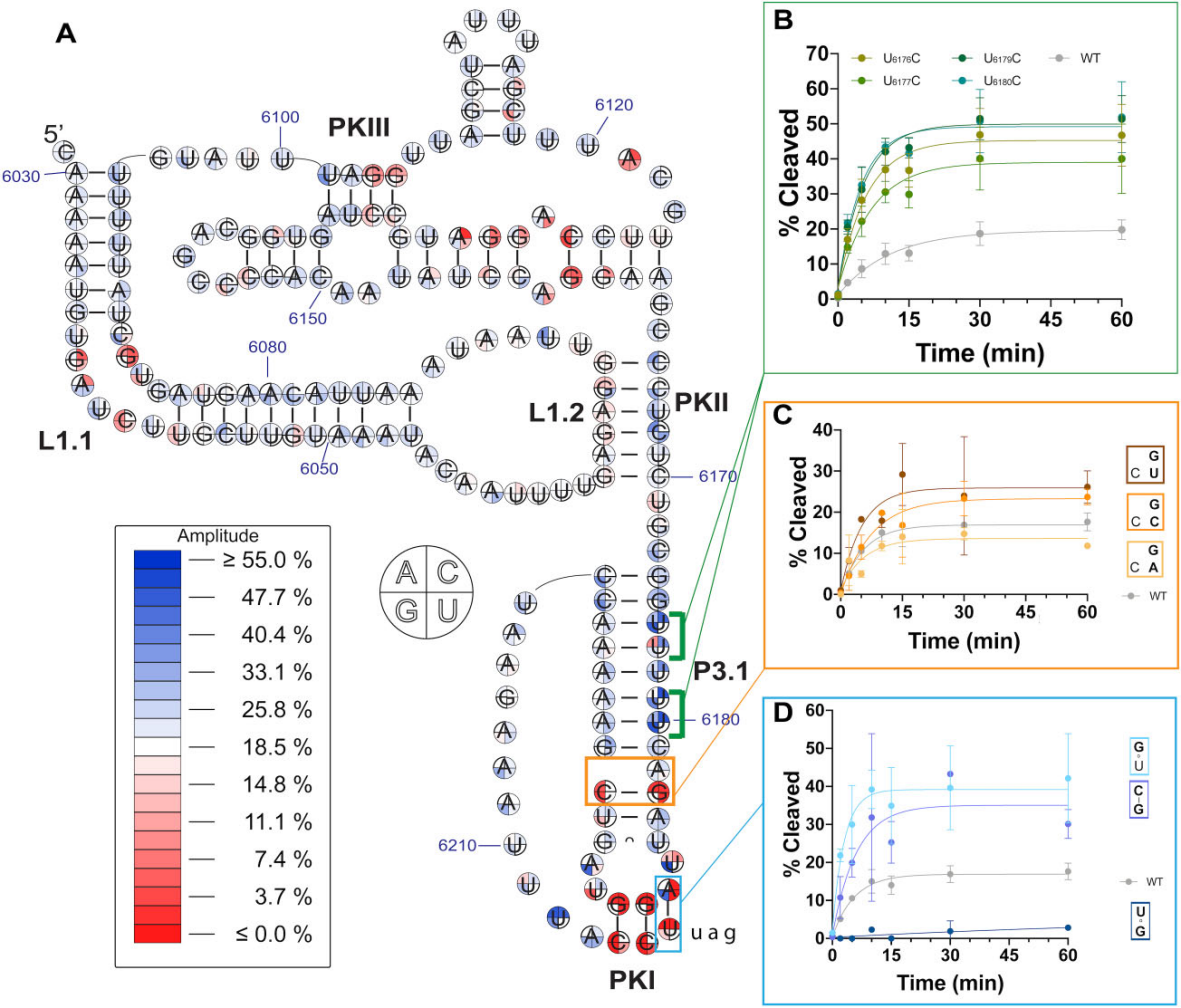
(**A**) Single mutant heatmap of average amplitude calculated from all replicate experiments. White represents wild-type amplitude. RelE cleavage traces are shown for (**B**) U-to-C mutations in P3.1, (**C**) double mutants involving the bulged position and C/G base pair, and (**D**) double mutants in PKI. Error bars in panels (B) through (D) reflect span of percent cleaved at that time point across replicates.

Ribosome loading capability as monitored by SMARTI is consistent with previously published CrPV IGR IRES data. Loop 1.1 is highly conserved and mediates essential contacts with the 60S subunit. Specifically, the mutants G_6038_C, C_6041_G, and G_6087_C have been shown to be deleterious [18]. These three mutants were included among the 81 198 mutants that were analyzed by SMARTI. Consistent with reported results, we observed that mutations at these positions reduced the amplitude and rate of IRES cleavage (Fig. 3A). The agreement in the loss of function caused by these mutations provides confidence in other mutants in the SMARTI dataset.

We saw further agreement with known mutational effects beyond ribosome binding contacts within PKI. These nucleotides mimic a codon/anticodon interaction that is essential for maintaining the IRES’s tRNA-like structure in this region. Previous activity studies reported that mutations to this pseudoknot, CC_6214–6215_GG, completely abrogate IRES activity [8, 14]. We observed a similar effect in the SMARTI analysis, which resulted in no cleavage after the 1-h incubation in wheat germ (Fig. 3A and Table 1). The observed consistency between published results and the SMARTI data establishes reliability in the data collected from novel mutants.

**Table 1.** Average fold change from wild type of amplitude and rate constant calculated from all replicate experiments that contained the IRES mutant

		Average fold change from wild type	
Region of IRES	Mutation	Amplitude	Rate constant
	Wild type	1	1
PKI	CC_6214–6215_GG	Not detectable	Not detectable
PKIII	U_6101_A	1.5 ± 0.1	1.0 ± 0.2
	A_6102_U	1.20 ± 0.03	1.1 ± 0.2
	UA_6101–6102_AU	1.1 ± 0.1	2.0 ± 1.8
	G_6103_C	0.5 ± 0.1	0.4 ± 0.2
	C_6134_U	1.0 ± 0.1	0.7 ± 0.1
PKII	C_6165_A	1.9 ± 0.1	1.0 ± 0.1
	CC_6165–6166_AG	1.7 ± 0.4	1.7 ± 1.1
	G_6066_U; C_6166_G	3.5 ± 0.4	1.0 ± 0.4
	C_6166_U	1.1 ± 0.1	1.2 ± 0.2

CC_6214–6215_GG not only was a useful comparison to prior literature, but also served as a helpful control. Based on the known CrPV mechanism, RelE should be unable to cut until after the first pseudotranslocation event removes PKI from blocking the A-site. We confirmed using our dataset that IRES RNA would not be cleaved unless the IRES accomplished 40S binding, 60S recruitment, and a successful pseudotranslocation. CC_6214–6215_GG has been shown capable of binding to the 40S subunit with similar affinity to wild type but unable to undergo pseudotranslocation [8]. Additionally, similar mutations disrupting PKI of the CrPV-like Platia stali virus IRES are still capable of binding to the 80S subunit [20]. Combined with the loss of function observed in our dataset, this evidence indicates that we see RNA cleavage upon completion of ribosome binding and eEF2-mediated pseudotranslocation in a manner consistent with previous mutational studies.

### Broken base pairs in PKI region enhance IRES ribosome loading

Due to the massive size of the dataset, we are unable to discuss all of the mutants in the collection. The entire data table for all 81 198 mutants is available in the Supplementary data. The table serves as a kind of consensus sequence of the CrPV IRES. In this section and those below, we report and discuss a subset of mutants that we thought warranted attention. We focus on the sequence requirements within the three pseudoknots of the IRES element. We anticipate that mutants we have analyzed outside these regions may be of interest to the field, though we have not discussed them here.

The region encompassing PKI is a tRNA-like domain that mimics a codon–anticodon interaction of a P/E hybrid state tRNA. This feature allows the IRES to initiate translation in the midst of an elongation cycle [16, 17]. It is well established that this pseudoknot is essential for IRES function and disruption of PKI results in a nonfunctional IRES [8, 14, 17, 20]. However, less is known about the helical portion of the hairpin leading into the pseudoknot, P3.1 [13]. One study looked at the impact of broad mutations that generated prolyl-tRNA-like IRES helices, as well as base pair deletions above the bulged nucleotide A_6182_. It showed that prolyl-tRNA and IRES chimeras of this region were less functional than the wild-type IRES and that the IRES was tolerant of a single base pair deletion above the bulged nucleotide [16].

Though this helix is known to be sequence sensitive, we were interested in how individual nucleotides contribute to the function of this IRES region using the fine single-nucleotide data from the SMARTI dataset. While the majority of mutants have near-wild-type amplitudes and rate constants, we identified some single mutants within the tRNA-mimicking domain that improve both the amplitude and rate constant of the mutants almost two-fold relative to wild type (Fig. 2B, labeled data points). We observed not only that the region above the bulged nucleotide A_6182_ accommodates mutations, but that ribosome binding and pseudotranslocation are enhanced by most single mutations to this region (Fig. 3A). Most notable were mutations that break base pairs in the string of uridine residues at positions 6176–6180, especially U-to-C mutations. U_6179_C and U_6180_C impart a two-fold increase in rate and more than a two-fold increase in amplitude as compared to wild type (Fig. 3B). These broken base pairs may provide increased flexibility in the helix that could enhance the IRES’s ability to load onto the ribosome. Moreover, flexibility may further help the IRES during pseudotranslocation. Nuclear magnetic resonance spectra and weak cryo-EM (cryogenic electron microscopy) density of this IRES region speak to its dynamic nature, and it was previously speculated that because this domain needs to move entirely out of the A-site during pseudotranslocation, innate flexibility allows for minimal disruption of ribosomal contacts [13, 17]. Given the length of this helix and the already large number of AU base pairs, it is fitting that it accommodates, and in fact favors, broken base pairs during ribosome binding and pseudotranslocation.

However, elsewhere in the helix maintaining a base pair is beneficial. The base pair between G_6183_ and C_6194_ is important for IRES function. All single mutations at either position, except those that retain a GU wobble pair, completely abrogate IRES initiation (Fig. 3A). The precise location of this base pair and the adjacent bulged nucleotide are seemingly modifiable. To explore this, we looked at double mutants in this region and observed that the G_6183_ single mutants are rescued by A_6182_G (Fig. 3C). A likely explanation for this observation is that A_6182_G can base pair with C_6194_ and the nucleotide at position 6183 is bulged instead of the one at 6182. Together, this indicates that the IRES can accommodate this base pair either above or below the bulged nucleotide and that the location and identity of the bulged nucleotide can be altered.

In addition to helix P3.1, we identified a mutation within PKI that enhances IRES function. The A_6187_G mutant generates a GU wobble in place of an AU pair in the position of PKI most proximal to the first codon. We observed an increase in both amplitude and rate constant for this mutant compared with wild type (Figs 2B and 3D). However, double mutation to create a GU wobble in the other direction, A_6187_U and U_6216_G, resulted in a nonfunctional IRES (Fig. 3D). Position 6187 makes stabilizing interactions with 18S RNA in the P-site and is conserved as a purine in canonical IGR IRESs making contacts with h18 in the A-site [7, 12, 45]. Our data show that a purine is not absolutely necessary in position 6187, as shown by the double mutant A_6187_C/U_6216_G (Fig. 3D). However, in the context of a noncanonical base pair like a GU wobble, the importance of a purine in this position for CrPV IRES is underscored as evidenced by the abrogated function of A_6187_U/U_6216_G. Both PKI and helix P3.1 exemplify a fine-tuned flexibility that aids the IRES in establishing binding interactions and handling the movement of pseudotranslocation.

### Pseudoknot III is composed of distinct expendable and critical base pairs

We also examined whether the other pseudoknots contained sequence elements that could be similarly modified. PKII and PKIII folding allows for correct positioning of accessory elements, SLIV, SLV, and Loop 1.1, that mediate essential contacts with the ribosomal subunits. Previous work, which mutated the entirety of PKIII, showed its importance for ribosome binding. That mutant reduced ribosome binding approximately four-fold compared to wild type [20].

The SMARTI results reiterate the importance of this region, but the single-nucleotide resolution of the data shows that within the 4-bp-long PKIII, two of the base pairs are important for ribosome loading while the other two are mutable. Base pairs U_6101_/A_6137_ and A_6102_/U_6136_ can be broken with any mutation and on either side while retaining the ability to bind to the ribosome and to be pseudotranslocated. Moreover, breaking both of these base pairs still results in an IRES with near-wild-type function (Fig. 3A and Table 1). To further understand the role of these base pairs, we examined structures that spanned the process of CrPV IGR IRES translation initiation. When comparing the 80S-bound IRES structure, 80S- and eEF2-bound IRES structure, and the 80S-bound and pseudotranslocated structure, a gradual opening of the U_6101_/A_6137_ base pair in PKIII is visible [12, 13, 15]. eEF2 binding seems to disrupt the contacts between the base-paired residues and upon pseudotranslocation, A_6137_ is shifted away from its original location in the helix. Given the inevitable disruption within the first position of the pseudoknot, it follows that mutations that break this base pair would be tolerated.

In contrast to the AU base pairs in PKIII, G_6103_/C_6135_ and G_6104_/C_6134_ are crucial for ribosome binding and pseudotranslocation. Almost all mutations that break either base pair result in loss of IRES function. C_6135_U or C_6134_U is able to restore function, which suggests that it is the location of the base pairs in this pseudoknot that is important and not the identity of the base pair (Fig. 3A and Table 1). Furthermore, compensatory mutations at these positions restore wild-type function signifying that sequence identity is not important for successful ribosome loading. Together, these data suggest that the paired structure of PKIII encompassing 6103–6104 and 6134–6135 is essential for ribosome loading and pseudotranslocation, while base identity is incidental. An alignment of CrPV-like IRES sequences showed that in all but one sequence, the last two base pairs of PKIII, equivalent to CrPV GG_6103–6104_/CC_6134–6135_, are GC base pairs [20]. GC base pair conservation at this location underscores the importance of these final two positions. Combining the SMARTI results with evolutionary conservation demonstrates that the IRES maintained GC base pairs at the critical positions in this pseudoknot.

### Pseudoknot II length and sequence are mutable

Given their shared importance in ribosome binding, we also explored whether PKII is amenable to as much modification as PKIII. A previous mutational study examining PKII showed that mutation of the entire pseudoknot resulted in an almost four-fold increase in 40S subunit *K*_D_ as compared with the wild-type IRES [8]. By SMARTI, we observed that PKII length could be seemingly reduced by one or two nucleotides with C_6165_A and CC_6165-6166_AG and maintain ribosome binding and pseudotranslocation ability (Table 1). However, PKII showed an asymmetric sensitivity to mutation. The loop 1.2 side of the helix was negatively impacted by mutation, whereas the other side of the helix showed modest amplitude increase as compared with wild type upon breaking base pairs (Fig. 3A). G_1064_ and A_1065_ interact with the large ribosomal subunit protein uL5, which could contribute to this asymmetry [7, 13]. Together with our data, this may indicate that while the IRES can function with reduced base pairing in the pseudoknot, there are nucleotide identity preferences for each side of the helix. This is underscored by the improved loading ability of GC_6066,6166_UG, which produced a three-fold amplitude increase as compared to wild type. When the orientation of the GU wobble in this position was reversed (mutant C_6166_U), the RNA functioned with wild-type activity (Table 1).

### Mutants’ ability to produce protein emphasizes complexity of IRES mechanism

**Figure 4.**
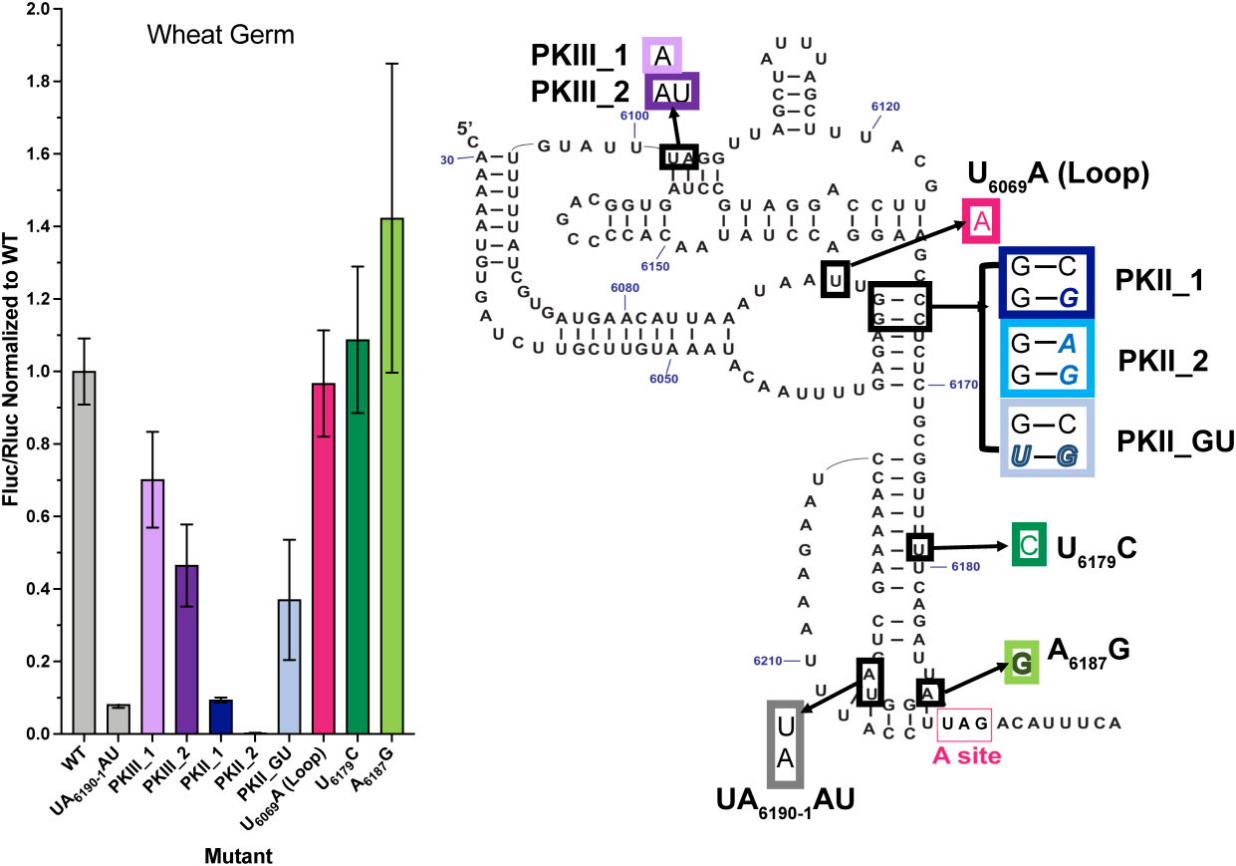
Protein production by selected mutants, identified on secondary structure diagram, was assessed using the dual-luciferase reporter assay. Shown are plots of average normalized Fluc/Rluc ratio from three independent replicates performed in wheat germ; error was propagated and is reflected in the error bars.

To test the ability of the improved mutants to translate protein, we utilized a dual-luciferase reporter system in wheat germ extract (Fig. 4). Translation of Fluc was under the control of the CrPV IGR IRES and production of Rluc was controlled by uncapped Kozak-sequence-mediated translation. The ratio of Fluc activity to Rluc activity provided a standardized measure of mutant IRES activity in promoting translation. We used a mutant known to abrogate IRES function, UA_6190–6191_AU, to serve as a negative control [17]. IRES sequences that improve function might be expected to produce more Fluc as compared to wild type and this would be reflected in an increased Fluc/Rluc ratio.

We assessed the function of selected mutants in three independent replicates of the dual-luciferase assay. Discussed below are data collected in wheat germ extract, although these trends were replicated in rabbit reticulocyte lysate (Supplementary Fig. S1C). The negative control, UA_6190–6191_AU, was more than 20-fold less functional than the wild-type IRES with a Fluc/Rluc ratio of 0.08 ± 0.01 as compared to 1.00 ± 0.09 for wild type.

To examine P3.1, we selected U_6179_C, one of the many U-to-C mutations that showed improved ribosome loading and pseudotranslocation by SMARTI. For PKI, we used A_6187_G, which generates a GU wobble in PKI and a similarly enhanced IRES. U_6179_C and A_6187_G achieved ratios of 1.09 ± 0.20 and 1.42 ± 0.43, respectively. A_6187_G therefore produced a minimal increase over wild-type protein production in wheat germ. The dampened impact of these mutants on protein production may suggest an additional role for these positions beyond the first pseudotranslocation event. Additionally, it is possible that these mutations may positively impact steps that are no longer rate-limiting in the context of producing full-length protein. A case for the latter exists if we consider the kinetics of downstream steps of translation, namely the translocation event after tRNA delivery. This second translocation event has been shown to occur at a rate comparable to the first pseudotranslocation event [44]. Therefore, the IRES sequences identified by SMARTI likely improve IRES function through pseudotranslocation but may still be limited by the downstream rate-limiting steps involved in translation.

We then tested mutants in PKIII of the IRES. By SMARTI, PKIII mutants that disrupted the first or first two base pairs of the pseudoknot retained near-wild-type function. In the translation assays, PKIII_1 and PKIII_2 produced ratios of 0.70 ± 0.13 and 0.46 ± 0.11, respectively. Therefore, PKIII can be shortened by half while maintaining a functional IRES. Combined with the SMARTI results, this clarifies which features of this pseudoknot have been evolutionarily conserved for their functional importance. An alignment of CrPV-like IRES sequences showed that each IRES contained a PKIII of greater than or equal to four base pairs [20]. We observed that this length is not required for IRES function but it may serve to fine-tune the IRES for the level of function that is required during infection.

Finally, we tested mutations to PKII that were shown by SMARTI to generate improved IRES sequences. We found that the mutation that generated a GU wobble at the second base pair in the helix, PKII_GU, was detrimental for protein production. This mutation of a CG base pair to a GU wobble reduced the protein production ability of the IRES about three-fold with an activity ratio of 0.37 ± 0.17. Additionally, other mutations to PKII, PKII_1, and PKII_2 that disrupted the first base pair or first two base pairs of the pseudoknot, respectively, were shown by SMARTI to have near-wild-type functionality, but were virtually unable to synthesize protein. Breaking a single base pair in PKII, PKII_1, resulted in an activity ratio of 0.09 ± 0.01 and breaking two base pairs, PKII_2, amounted to an almost undetectable amount of protein. Similarly, we tested an improved mutant, as assessed by SMARTI, in loop 1.2, U_6069_A (loop). This mutant did not produce a significant difference from wild type in protein production resulting in a ratio of 0.96 ± 0.15.

Taken together, while PKII is known to play a role in ribosome binding, these results suggest an additional role for this region that occurs after the first pseudotranslocation. These mutations, which seem to enhance ribosome binding or pseudotranslocation, may have a detrimental effect on the subsequent process of tRNA delivery, frame selection, or next translocation events. However, it is important to remember that CrPV IGR IRES must balance between being stable and dynamic as it forms the structure and ribosomal contacts required for translation initiation [46]. The additional base pairs in PKII may be important in maintaining the correct orientation of the IRES and in preserving contacts both within the IRES and between the IRES and ribosome as it moves.

### Accessibility and applicability of SMARTI enhance bioengineering

The use of this high-throughput mutational analysis has allowed us to refine the understanding of a well-studied IRES sequence. Previous studies on the CrPV IGR IRES mutated several bases at a time, or even whole helices, in order to delineate defined roles for each domain and enable directed mutational screens to identify some key nucleotides. With this extensive dataset, we are able to see the impact of all individual mutations that serves as a resource for future analysis of IRES function. We have shown that this high-throughput sequencing-based approach using RelE cleavage as a readout for A-site binding is compatible with eukaryotic extract. Extract and lysate compatibility provides an avenue for studying diverse eukaryotic RNA elements that require more protein factors, for examining RNA function in a more native environment, and for increasing the utility of the SMARTI assay by eliminating the need for purified components.

Such a fine-level single-mutation screen additionally offers a powerful approach to identify improved RNA sequences that can be used as biological tools. The current study presents several mutations that improve the IRES at the level of binding and pseudotranslocation. Identifying short and improved engineered sequences for producing protein is an additional valuable pursuit and other IRESs offer a wide array of targets as part of that goal. Application of this method will advance our understanding of the biology, evolution, and engineering potential of these sequences.

## Supplementary Material

gkaf445_Supplemental_Files

## Data Availability

Raw sequencing data analyzed in this manuscript is deposited in the Sequencing Read Archive (BioProject ID: PRJNA1219037). An Excel file containing all the fit parameters for the single and double mutants is included in the supplementary data. Any additional data pertinent to this work is available from the author upon request.
