# Supplementary material for: Comprehensive mutational analysis of the sequence–function relationship within a viral internal ribosome entry site
Source: Nucleic Acids Res. 2025 May 27;53(10):gkaf445. doi: 10.1093/nar/gkaf445 (PMC12107430; doi:10.1093/nar/gkaf445)
Supplement: gkaf445_Supplemental_Files [file gkaf445_supplemental_files.zip › Supplemental_Information_Figures.pdf]

Supplementary Information

Comprehensive mutational analysis of the sequence-function relationship within a viral internal ribosome entry site

Sabrina G. Grunseich and Scott A. Strobel

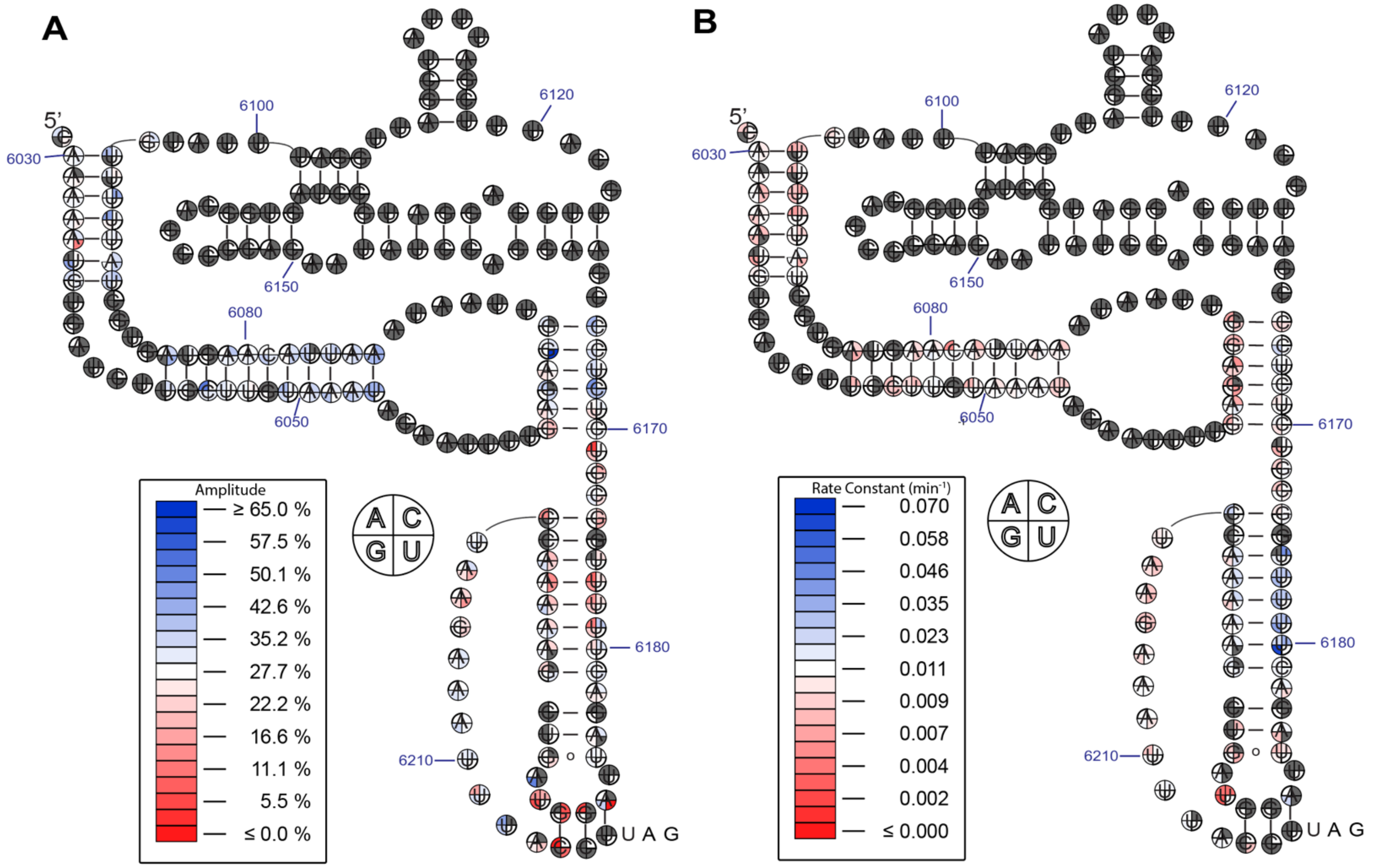

Supplemental Figure 1: Single mutant heat map of (A) amplitude and (B) rate constant from one rabbit reticulocyte lysate experiment using the PKI library. Amplitudes were excluded if their standard deviation was greater than 10 percentage points. Rate constants were excluded if the corresponding amplitude was excluded, the amplitude was less than 5% cleaved, or the standard deviation was greater than the value. White represents wild-type amplitude. Gray represents excluded values or those not included in the library. (C) Protein production assessed using the dual luciferase reporter assay by selected mutants (identified on secondary structure diagram in Figure 4). Shown is a plot of average normalized Fluc/Rluc ratio from three independent replicates performed in rabbit reticulocyte lysate, error was propagated and is reflected in the error bars.

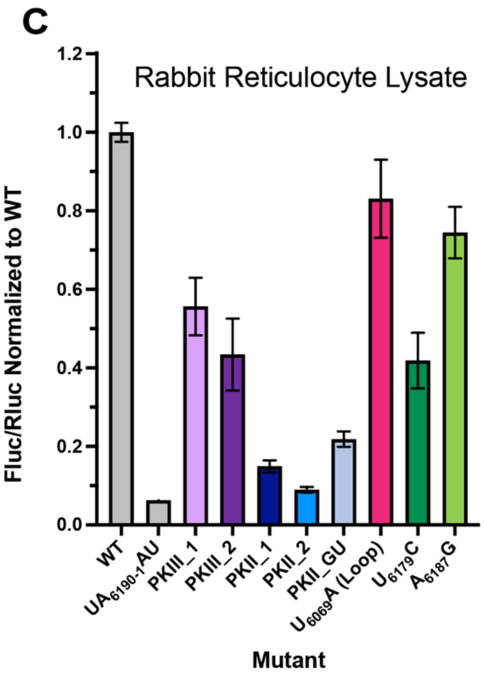

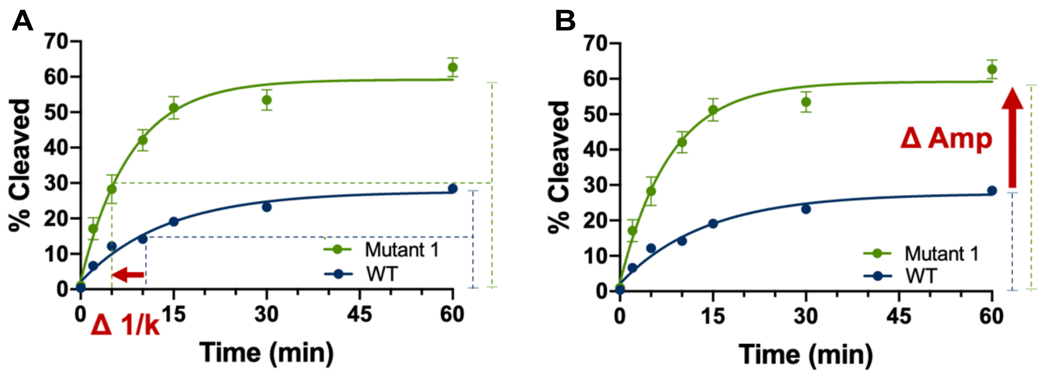

Supplemental Figure 2: Representative curve indicating a shift in (A) rate constant, k, and (B) amplitude.

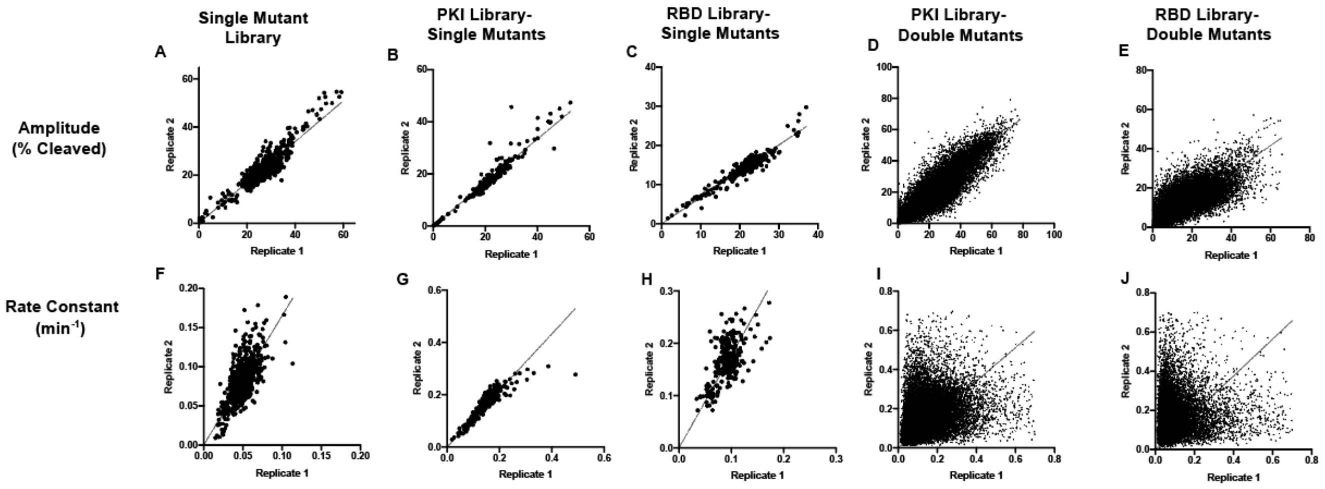

Supplemental Figure 3:

Correlation of mutant amplitude (top row) and rate constant (bottom row) between replicates. Columns differ by library and number of mutations per mutant.
